# A Monoclonal Anti-HMGB1 Antibody Attenuates Neurodegeneration in an Experimental Animal Model of Glaucoma

**DOI:** 10.3390/ijms23084107

**Published:** 2022-04-07

**Authors:** Henrik Tonner, Selina Hunn, Nadine Auler, Carsten Schmelter, Vanessa M. Beutgen, Harald D. von Pein, Norbert Pfeiffer, Franz H. Grus

**Affiliations:** 1 Department of Experimental and Translational Ophthalmology, University Medical Center, Johannes Gutenberg University, Langenbeckstr. 1, 55131 Mainz, Germany; htonner@eye-research.org (H.T.); selinahunn@web.de (S.H.); nauler@eye-research.org (N.A.); cschmelter@eye-research.org (C.S.); vbeutgen@eye-research.org (V.M.B.); norbert.pfeiffer@unimedizin-mainz.de (N.P.); 2 Institute of Neuropathology, University Medical Center, Johannes Gutenberg University Mainz, 55131 Mainz, Germany; highlander1@freenet.com

**Keywords:** glaucoma, antibodies, neuroprotection, neurodegeneration, discovery proteomics, microarray, animal model

## Abstract

Neuroinflammation is a crucial process for the loss of retinal ganglion cells (RGC), a major characteristic of glaucoma. High expression of high-mobility group box protein 1 (HMGB1) plays a detrimental role in inflammatory processes and is elevated in the retinas of glaucoma patients. Therefore, this study aimed to investigate the effects of the intravitreal injection of an anti-HMGB1 monoclonal antibody (anti-HMGB1 Ab) in an experimental animal model of glaucoma. Two groups of Spraque Dawley rats received episcleral vein occlusion to chronically elevate intraocular pressure (IOP): (1) the IgG group, intravitreal injection of an unspecific IgG as a control, *n* = 5, and (2) the HMGB1 group, intravitreal injection of an anti-HMGB1 Ab, *n* = 6. IOP, retinal nerve fiber layer thickness (RNFLT), and the retinal flash response were monitored longitudinally. Post-mortem examinations included immunohistochemistry, microarray, and mass spectrometric analysis. RNFLT was significantly increased in the HMGB1 group compared with the IgG group (*p* < 0.001). RGC density showed improved neuronal cell survival in the retina in HMGB1 compared with the IgG group (*p* < 0.01). Mass spectrometric proteomic analysis of retinal tissue showed an increased abundance of RNA metabolism-associated heterogeneous nuclear ribonucleoproteins (hnRNPs), such as hnRNP U, D, and H2, in animals injected with the anti-HMGB1 Ab, indicating that the application of the antibody may cause increased gene expression. Microarray analysis showed a significantly decreased expression of C-X-C motif chemokine ligand 8 (CXCL8, *p* < 0.05) and connective tissue growth factor (CTGF, *p* < 0.01) in the HMGB1 group. Thus, these data suggest that intravitreal injection of anti-HMGB1 Ab reduced HMGB1-dependent inflammatory signaling and mediated RGC neuroprotection.

## 1. Introduction

Glaucoma comprises a group of neurodegenerative diseases, with the most prevalent form being primary open-angle glaucoma (POAG). The disease progression is defined as the slow but steady and irreversible death of retinal ganglion cells (RGCs), which results in progressive loss of vision and ultimately leads to the complete blindness of the patients [1]. Elevated intraocular pressure (IOP) is considered the main risk factor and the only treatable symptom of glaucoma. However, despite successful IOP-lowering therapy, the disease may progress, which underlines glaucoma as a multifactorial disease. Considering that only 70% of glaucoma patients show an elevated IOP, it seems evident that different molecular mechanisms, including oxidative stress, glutamate toxicity, and vascular dysfunction, may cause the degeneration of RGCs. These risk factors might also play a role in disease progression, besides an elevated IOP [2,3].

In recent years, strong evidence has been provided that glaucoma has an autoimmune component in addition to the known pathophysiology. In glaucoma patients, autoantibody deposits appear in the retina [4,5,6]. This was a surprising finding, since the eye was considered an immune-privileged organ [7]. The expression of different autoantibodies (Aabs), also called autoantibody profiles, plays a crucial role in glaucoma. These profiles were altered in the serum and aqueous humor of glaucoma patients [8,9,10]. Upregulated Aabs, for example, anti-HSP 27 Aabs, have been frequently found in glaucoma patients and were characterized as autoaggressive, while downregulated Aabs, for example, against anti-α-synuclein Aabs, have been hypothesized to be neuroprotective [9,11,12,13]. The neuroprotective effects of antibodies (Abs) have been demonstrated in cell culture, retinal organ culture, and glaucoma animal models [14,15,16,17], indicating the potential of recombinant Abs in an immunotherapeutic approach for glaucoma treatment.

It has been shown that an inflammatory response enhances neurodegenerative processes in many diseases, including autoimmune diseases such as Alzheimer’s, Parkinson’s, or systemic lupus erythematosus (SLE) [7,18,19,20,21]. A pro-inflammatory milieu is also present in glaucoma. Autoantibodies may play a crucial role, as it has already been shown that autoantibodies can influence cytokine expression levels such as CXCL8 (alternative name: IL-8) and TNF-α [22].

The high-mobility group box protein 1 (HMGB1) is a versatile non-histone-binding protein, which mainly acts under physiological conditions as a DNA chaperone in the nucleus. It is released into the cytoplasm during inflammatory responses and acts as an autophagy-regulating protein [23,24]. In the extracellular milieu, HMGB1 is considered a prototypic damage-associated molecular pattern molecule (DAMP) [25]. It can be actively secreted by apoptotic or necrotic neurons by exocytosis or passively and then interacts as a pro-inflammatory cytokine or ligand with various receptors such as Toll-like receptors 2 and 4 (TLR-2, TLR-4). The receptor for advanced glycation endproducts (RAGE) also showed a high affinity for extracellular HMGB1 and can prolong the inflammatory cell response upon activation [26]. This has already been demonstrated for Alzheimer’s disease, Parkinson’s disease, and Sjörgen’s syndrome, among others [27]. Interestingly, patients who tested positive for anti-HMGB1 Aabs after septic shock were predicted to have a better chance of recovery than anti-HMGB1-negative patients [28].

This study aimed to investigate the effects of the anti-HMGB1 Ab in a model of chronic IOP elevation. For this purpose, IOP, retinal nerve fiber layer thickness (RNFL), and an electroretinogram (ERG) were recorded in vivo. In addition, RGC density and axon damage in the optic nerve head (ONH) were assessed in post mortem analyses. To investigate the possible molecular effects of anti HMGB1 Ab application, microarray and mass spectrometric analyses of retinal tissue were conducted. We hypothesize that the anti-HMGB1 Ab treatment reduces the target protein extracellularly, which could reduce the ability of HMGB1 to bind receptors such as TLR-2, -4, or RAGE. This could reduce IOP-induced pro-inflammatory stimulation, resulting in less death of RGCs.

## 2. Results

### 2.1. Chronic IOP Elevation by Episcleral Vein Occlusion

**Figure 1:**
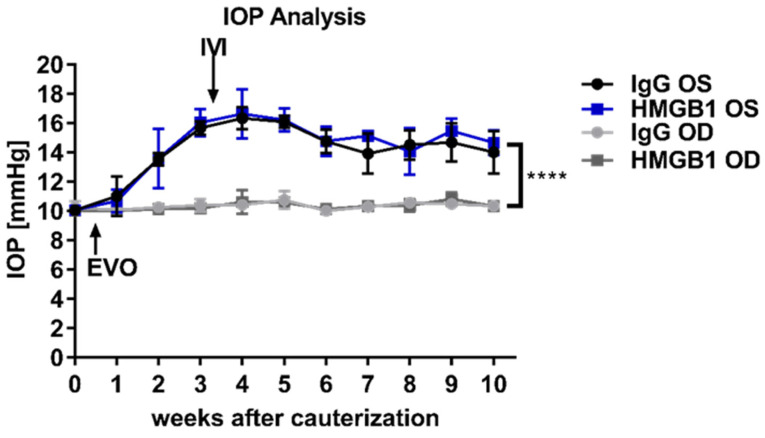
Follow-up of IOP elevation. IOP was measured once a week using TonoLab. After episcleral vein obliteration (EVO), IOP increased to a stable level within three weeks. After achieving a stably elevated IOP, intravitreal injection (IVI) was performed into OS. **** *p* < 0.0001, Student’s *t*-test.

IOP elevation was performed unilaterally on the left eye (OS) to induce glaucoma-like damage; the right eye (OD) served as an internal control. After stable IOP elevation, one group received IgG control antibodies (IgG OS group) or an anti-HMGB1 Ab (HMGB1 OS group) in the respective OS. Intraocular pressure increased to 15.7 ± 0.5 mmHg in the IgG OS group and 16.0 ± 0.9 mmHg in the HMGB1 OS group three weeks after EVO (Figure 1). Over the further observation period of the study, IOP remained significantly elevated until week 10 after EVO; IOP was 14.0 ± 1.5 mmHg in the IgG OS and 14.7 ± 0.8 mmHg in the HMGB1 OS group, respectively (Student’s *t*-test, *p* < 0.0001). The IOP in the OD of the respective groups did not change significantly. The measurements were 10.3 ± 0.3 mmHg in the IgG OD and 10.3 ± 0.1 mmHg in the HMGB1 OD group.

### 2.2. Anti-HMGB1 Antibodies Attenuate RGC Loss

**Figure 2:**
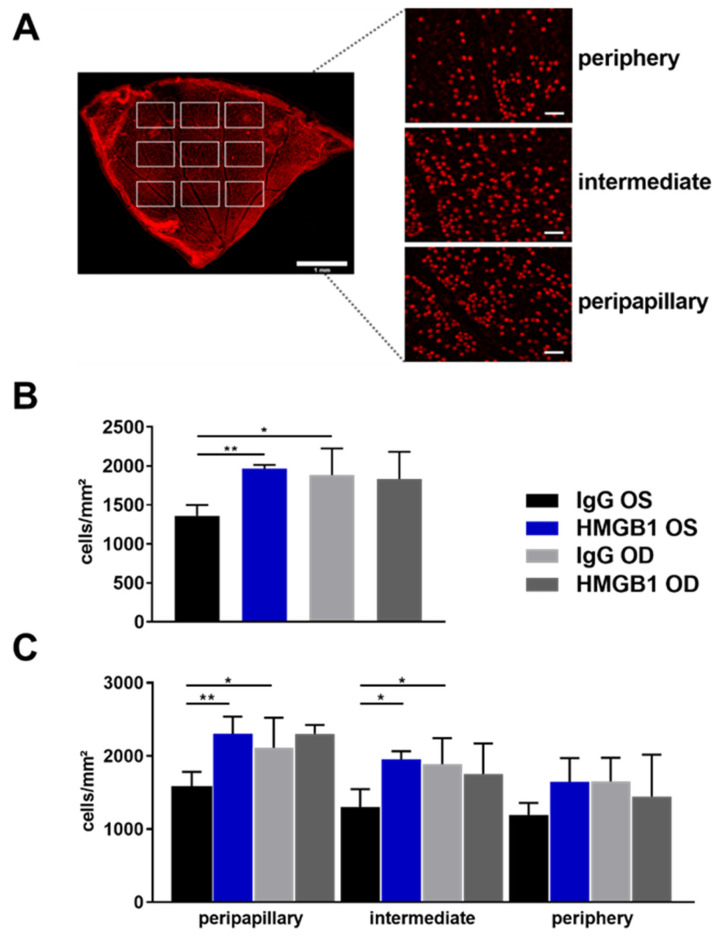
Immunostaining and quantitative analysis of Brn3-positive RGCs in retinal flat mounts. The increase in IOP-induced neurodegenerative processes, including retinal ganglion cell death. Immunostaining of RGCs from a quarter of a retinal flat mount by the cell marker Brn3a was used to quantify the surviving RGCs. Nine images were obtained from this quarter, three images each from one region: peripapillary, intermediate, and periphery (**A**, scale bar: 1 mm). To calculate the RGC density, the mean value of the RGC number of all nine images was used (**B**, scale bar: 50 µm). For the regional RGC density analysis, the mean value of the RGC number of the three images of a region was used (**C**). Finally, the calculated RGC numbers were related to the area of one square millimeter. For staining, an anti-Brn3a antibody (C20, Santa Cruz) was used as the primary antibody, and an anti-goat AF568 (Life Technologies) was used as the secondary antibody. The representative overview image of a retinal quarter was taken with a confocal microscope (Leica TCS SP5 confocal microscope, 10 × 0.3 Air objective, IMB Microscopy Core Facility, Mainz, Germany); scale: 1 mm. Single images were taken with a fluorescence microscope (Eclipse TS 100 microscope, Nikon, Yurakucho, Tokyo, Japan) DS-Fi1-U2 digital microscope camera (Nikon). Objective: ELWD 20×/0.45 S Plan Flour Ph1 ADM Air objective (Nikon). * *p* < 0.05, ** *p* < 0.01, not significant (ns), one-way ANOVA, Tukey’s HSD post hoc test.

To quantify the glaucomatous damage on RGCs, immunostaining of the reliable RGC marker brain-specific homeobox/POU domain protein 3A (Brn3a) was performed. One-quarter of the retina was used and examined in nine images for staining. Three images were taken in each region, peripapillary, intermediate, or peripheral, according to the distance of the optic nerve head (ONH, Figure 2A). The mean RGC density of the total flat mount was 1359 ± 140 cells/mm^2^ in the IgG OS group and significantly lower compared with 1968 ± 46 cells/mm^2^ and 1885 ± 341 cells/mm^2^ in the HMGB1 OS and IgG OD groups, respectively (Figure 2B, * *p* < 0.05, ** *p* < 0.01). The RGC density in the HMGB1 OD group was 1833 ± 349 cells/mm^2^, which was not significantly changed compared with the HMGB1 OS group. The difference in RGC density between HMGB1 OS and IgG OS was 31%, and the difference between IgG OS and IgG OD was 28%.

A closer look at the regional RGC density showed that the RGC density revealed region-specific differences between the IgG OS and the HMGB1 OS group (Figure 2C). In the central area, the RGC density in the IgG OS group (1577 ± 207 cells/mm^2^) was significantly lower in comparison with the HMGB1 OS group (2304 ± 236 cells/mm^2^, *p* < 0.01). The RGC density of IgG OS compared with the IgG OD group (1653 ± 324 cells/mm^2^) was also significantly lower (*p* < 0.05), while no significant differences were observed between the HMGB1 OS and HMGB1 OD (2302 ± 236 cells/mm^2^) groups.

In the intermediate region, the difference in the RGC density of the IgG OS group (1305 ± 242 cells/mm^2^) compared with the HMGB1 OS group (1953 ± 113 cells/mm^2^) was significantly decreased (*p* < 0.05), as well as compared with the IgG OD group (1888 ± 357 cells/mm^2^, *p* < 0.05). The RGC density of the HMGB1 OS group compared with the HMGB1 OD group (1754 ± 417 cells/mm^2^) was not significantly changed in the intermediate region.

The RGC density between the IgG OS and the HMGB1 OS group in the peripheral region is not statistically different. In this region, the RGC density in the IgG OS group is 1193 ± 165 cells/mm^2^. The RGC density in the HMGB1 OS group is 1647 ± 325 cells/mm^2^.

### 2.3. Administration of the HMGB1 Antibody Reduces the RNFLT Loss

**Figure 3:**
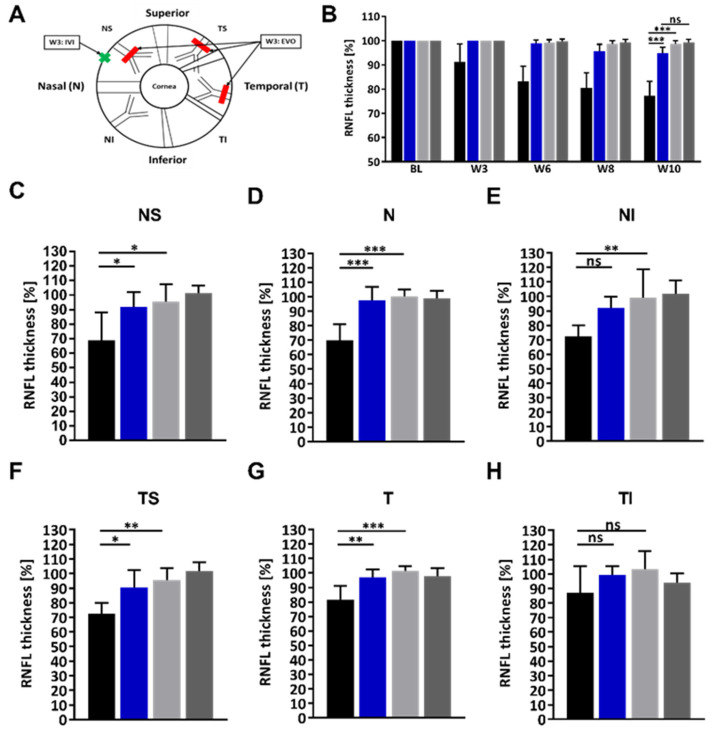
Quantification of the retinal nerve fiber layer thickness (RNFLT). To quantify the damage to the retina caused by the chronically elevated IOP in vivo, the retina was examined by optical coherence tomography (OCT). This allows retinal cross-sections to be imaged. The retinal nerve fiber layer was defined and quantified using a semi-automatic segmentation algorithm. For quantification, a 12° diameter circular B-scan was acquired. The Heidelberg Eye Explorer software divides the retina into the main sectors, superior (S), temporal (T), inferior (I), and nasal (N), as well as into the intermediate sectors, temporal superior (TS), temporal inferior (TI), nasal inferior (NI), and nasal superior (NS) (**A**). The scheme shows the interventions performed during the study on the eye, episcleral vein occlusion (EVO), and intravitreal injection (IVI) (scheme modified from [29]). The mean RNFLT of the whole B-scan was used for follow-up quantification (**B**). Further quantification of the RNFLT at week 10 was performed for the individual sectors (**C–H**). Legend: black (IgG OS), blue (HMGB1 OS), light grey (IgG OD), dark grey (HMGB1 OD). * *p* < 0.05, ** *p* < 0.01, *** *p* < 0.001, not significant (ns), one-way ANOVA, Tukey’s HSD post hoc test.

To quantify the IOP-induced damage to the retinal axons in the RNFL, retinal cross-sections were recorded using OCT. Analyzing the OCT images, the semi-automatic algorithm calculates the RNFLT as an overall average over six regions. Furthermore, the algorithm calculates the RNFLT for the nasal superior (NS), nasal (N), nasal inferior (NI), temporal superior (TS), temporal (T), and temporal inferior (TI) regions individually (Figure 3A).

Over the entire study period and all regions calculated together, there was a greater loss in the IgG OS group than the HMGB1 group. At week ten, RNFLT in the IgG OS group was still 77.3 ± 5.9% compared with baseline, whereas in the HMGB1 OS group RNFLT was significantly higher with 94.8 ± 2.4% (Figure 3B, *p* < 0.001). The difference in RNFLT of the IgG OS compared with the IgG OD group with 96.7 ± 1.2% was significantly lower at week ten (*p* < 0.001).

For the NS region (Figure 3C), RNFLT at week ten relative to the baseline is 68.9 ± 19.2%, significantly lower in the IgG OS compared with 91.9 ± 10.1% in the HMGB1 OS group (*p* < 0.05), as well as compared with the IgG OD group (*p* < 0.05).

In the nasal region (N, Figure 3D), RNFLT is 69.8 ± 11.2% in the IgG OS group compared with the HMGB1 OS group at 97.6 ± 9.3% (*p* < 0.001). The RNFLT in the IgG OD is 100.4 ± 4.4% which is significantly higher than the IgG OS (*p* < 0.001). The RNFLT of HMGB1 OD is 98.8 ± 5.4%, which is not substantially different from the HMGB1 OS.

In the nasal inferior region (NI, Figure 3E), the RNFLT of the IgG OS is 72.4 ± 7.7% at week 10 relative to the baseline measurement. In the HMGB1 OS group, it is 92.1 ± 7.5%, which is not significantly different. At week ten, the RNFLT of the IgG OD group was 99.3 ± 19.3% and significantly higher than the IgG OS group (*p* < 0.01). The RNFLT in the HMGB1 OD group was 101.9 ± 9.0% and was not very different compared with the HMGB1 OS group.

Looking at the RNFLT on the temporal side of the retina, the RNFLT for the temporal superior region (TS, Figure 3F) was 72.6 ± 7.3% in the IgG OS group and 90.3 ± 12.1% in the HMGB1 OS group compared with the respective baseline measurement (*p* = 0.02). The RNFLT in the IgG OD was 95.6 ± 8.1%, which is significantly different from the IgG OS group (*p* < 0.02). The difference between the HMGB1 OS group was not substantially different from the HMGB1 OD group, with 101.9 ± 5.7%.

In the temporal region (T, Figure 3G), RNFLT at week ten was 81.6 ± 9.3% in the IgG OS group and 97.1 ± 5.4% in the HMGB1 OS group (*p* < 0.01). IgG OD at week ten was significantly different, with RNFLT 101.5 ± 3.3%, compared with the IgG OS group (*p* < 0.01). At week ten, the HMGB1 OD group had an RNFLT of 97.9 ± 5.6%, which was not significantly different from the HMGB1 OS group.

In the temporal inferior (TI, Figure 3H) region, no significant changes could be detected.

Comparing the RNFLT differences in the regions, the difference in the RNFLTs of the IgG OS related to the HMGB1 OS group was more minor on the temporal side, with 17.7%, 15.5, and 12.3% (TS, T, and TI), than on the nasal side, with 22.5%, 27.8%, and 19.7% (NS, N, and NI) (Table 1). 

In the IgG group, the RNFLT in the nasal regions was comparable, with 68.9%, 69.8%, and 72.4% (NS, N, NI), whereas the RNFLT in the temporal areas was quite obviously different, with 72.6%, 81.6%, and 87.1% (TS, T, and TI). For the HMGB1 OS group, the RNFLT in the nasal side regions was approximately comparable in TS and TI, at 91.9% and 92.1%, respectively, whereas the RNFLT in N was higher at 97.6%. For the temporal side in the HMGB1 OS group, the RNFLT in the upper TS region was significantly lower at 90.3%, compared with the middle T region at 97.1% and the lower TI region at 99.4%. For the effects of anti-HMGB1 application on RNFLT, in summary, Ab significantly reduced the average loss of RNFLT over the entire observation period. Furthermore, for the regional observation, the difference in RNFLT between HMGB1 OS and IgG OS was greater on the nasal side than on the temporal side. Therefore, the greatest positive effect of the anti-HMGB1 application was seen for the regions NS and N.

### 2.4. Analysis of Retinal Functionality by Photopic Ganzfeld ERG

**Figure 4:**
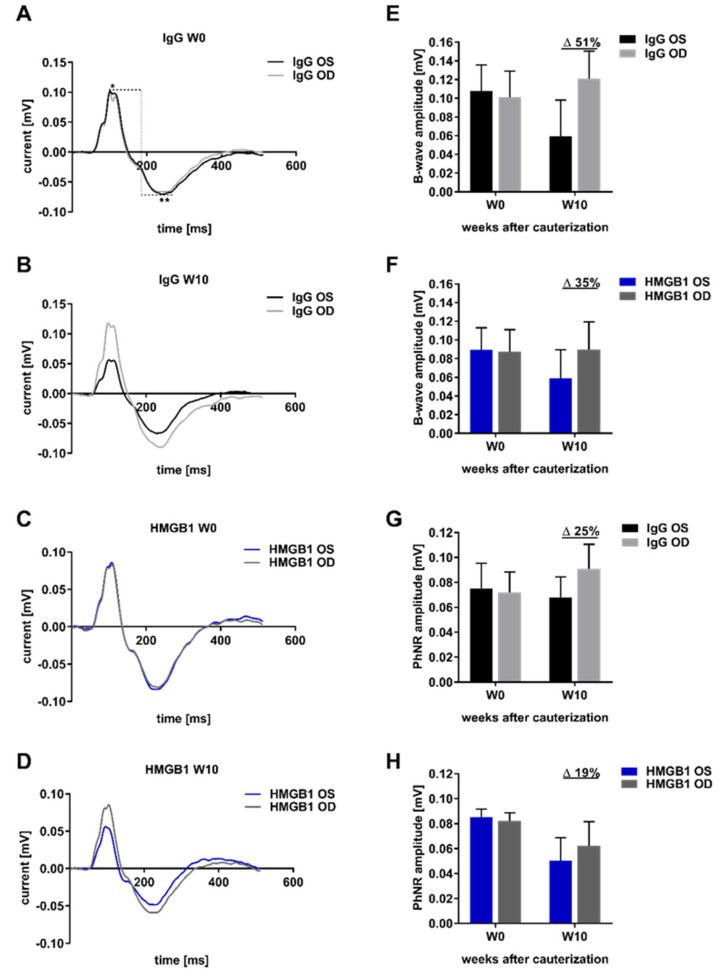
Ganzfeld electroretinogram (ERG) pattern and quantification of B-wave and PhNR amplitude at week 0 and week 10. ERG recordings were acquired at different time points. The mean values of the ERG recordings of the individual animals were plotted for the time points week 0 (before EVO) and week 10 after EVO (**A**–**D**). The flash intensity 1.37 log_10_ cd·s·m^−2^ was used for the ERG recordings shown. The B-wave amplitude (*) and the amplitude of the photopic negative response (PhNR, **) were used for quantification. Quantification was performed separately for the B-wave amplitude of the IgG (**E**) and the HMGB1 group (**F**) as well as for the PhNR amplitude of the IgG (**G**) and the HMGB1 group (**H**). The millivolt decrease was calculated as a percentage difference (Δ = % change).

For the analysis of IOP-induced damage on retinal functionality, the Ganzfeld electroretinogram (ERG) was used in this study. The ERG pattern showed remarkable changes at week 10 after EVO (Figure 4B,D) compared with week 0 (Figure 4A,C). The amplitudes of the B-wave and the photopic negative response (PhNR) were quantified at week 0 and week 10 after EVO. In the IgG OS group, the B-wave amplitude difference in OS related to OD was 51% at week ten (Figure 4E). This difference was less pronounced in the HMGB1 OS animals than in the IgG-injected animals, with 35% at week ten (Figure 4F). The difference in the PhNR amplitude in the IgG injected animals was 25% at week ten (Figure 4G). In the HMGB1 group, the difference was 19% at week 10 (Figure 4H). In summary, the measurement of retinal functionality showed that the anti-HMGB1 Ab application had a stronger positive effect on the preservation of B-wave amplitude than on PhNR amplitude. Therefore, the applied Ab has an effect not only on the RGCs but also on inner retinal layers, which respond mainly to the B-wave.

### 2.5. The HMGB1 Antibody Reduces the Grade of Optic Nerve (ON) Damage

**Figure 5:**
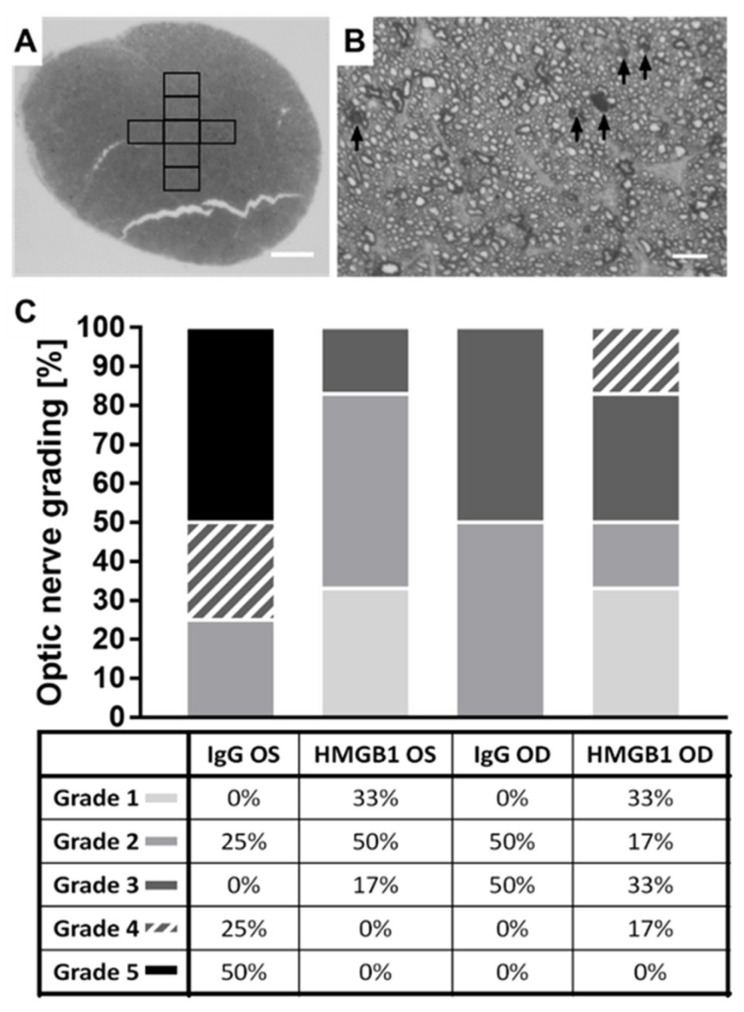
Grading of optic nerve cross-sections. The increased IOP causes damage to the axon bundles, the degree of which was analyzed by the number of collapsed axons in PPD-stained cross-sections of the optic nerves. An overview image was acquired using the 10× objective (**A**, scale bar: 100 µm). Seven tile scans from different areas of the optic nerves were acquired using the 100× oil objective (**B**, scale bar: 10 µm). The number of collapsed axons was determined. Grades were determined based on the highest sum of collapsed axons found in the 7 images (**C**). The sections were imaged with an upright microscope and a 100× immersion oil objective (Olympus Vanox-T AH-2; Olympus SPlan 100×/1.25 oil, 160/0.17, Olympus Deutschland GmbH, Hamburg, Germany).

To investigate the effect of the anti-HMGB1 Ab application on the optic nerve (ON), corresponding cross-sections were stained with *p*-phenylenediamine (PPD), and seven images were acquired (Figure 5A). The completely collapsed axons in each image were counted and summed for seven images of each nerve (Figure 5B, Black arrows). This resulted in a damage score for each ON. The results are presented as a stacked plot (Figure 5C). In the IgG OD group, 100% of the ON examined were grade 2 or 3. In the HMGB1 OD group, 83% of the optic nerves examined were grade 1–3. Therefore, the damage in the IgG OD and HMGB1 OD groups was approximately similar. For the IgG OS group, 75% of the evaluated ON showed a damage grade of 4 or 5. In the HMGB1 group, no ON had a damage grade of 4 or 5. In this group, 83% of the optic nerves were grade 1 or 2, indicating improved ON damage with anti- HMGB1 Ab application.

### 2.6. Proteomic Analysis

**Figure 6:**
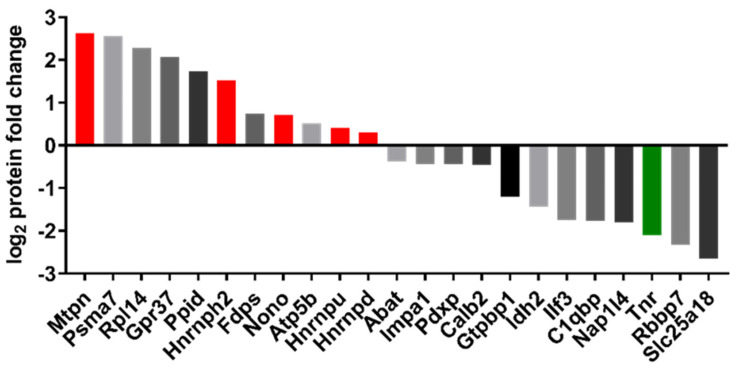
Illustration of the up- and downregulation of significantly altered protein expression identified by mass spectrometric analysis. Proteins were first isolated from the tissue after IOP elevation for proteomic analysis by mass spectrometry and then tryptically digested. The resulting peptides were purified for MS analysis. Peptides were identified, quantified, and normalized using MaxQant. For statistical analysis, a student’s *t*-test was used. The graph shows the upregulated and down-regulated proteins in the antibody-treated group compared with the control group. Of note were the three heterogeneous nuclear ribonucleoproteins that were upregulated and myotrophin (red). The extracellular matrix protein tenascin-R (Tnr), which was downregulated (green), also aroused great interest. The full names of the proteins are shown in Appendix A (Table A1).

Mass spectrometry and antibody-based microarray were used to identify molecular changes in the retina. These changes were used to derive molecular mechanisms and altered signaling pathways affected by the anti-HMGB1 Ab treatment. The mass spectrometric analysis revealed 747 retinal proteins, of which 23 were significantly altered (Figure 6, 11 low and 12 high abundance, *p* < 0.05). Most strikingly, many ribonucleic complex proteins are highly abundant, such as heterogeneous nuclear ribonucleoproteins (hnRNPs). Additionally, the protein myotrophin (Mtpn), involved in actin cytoskeleton formation, was highly abundant in the group treated with the anti-HMGB1 Ab. Tenascin-R (Tnr), a protein located in the extracellular matrix, was less abundant in the anti-HMGB1 Ab-treated eyes.

**Figure 7:**
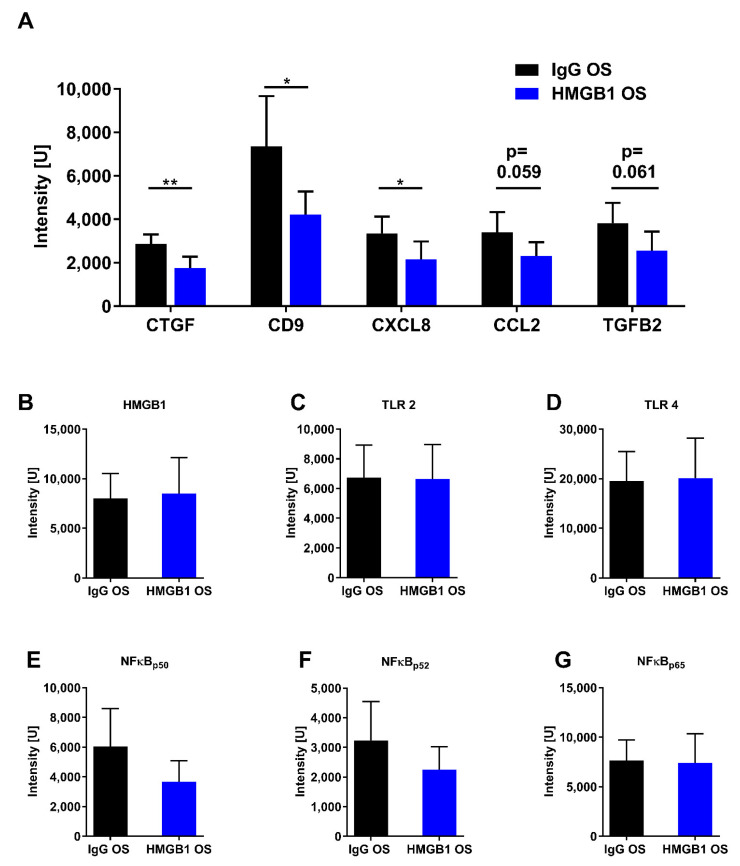
Expression pattern of selected retinal proteins analyzed by an antibody microarray. For the analysis, 10 µg of protein lysate was used. This was labeled, and a size exclusion chromatography column removed excess unbound dye and buffers unfavorable for the array incubation. The altered proteins with respective expressions in the elevated-IOP eyes were shown. ** *p* < 0.01, * *p* < 0.05, other *p* values as indicated, Student’s *t*-test (**A**). The protein expression of the high-mobility group box protein 1 (HMGB1) was examined in the IgG animals (OS, OD) and the antibody-treated HMGB1 animals (OS, OD) (**B**–**G**).

Given that HMGB1 plays a critical role in the inflammatory response, we also focused on cytokines and other pro-inflammatory proteins in this study. We used an antibody-based microarray to analyze protein abundances. Proteins such as CXCL8, connective tissue growth factor (CTGF), and CD9 were significantly less abundant in the HMGB1 OS group than in the IgG OS group (Figure 7A). Other proteins associated with a pro-inflammatory effect or inflammatory signaling pathways tended to be downregulated in the HMGB1 OS group compared with the IgG OS group, such as CCL2 (*p* = 0.059) and TGFβ2 (*p* = 0.061, Figure 7A), but they did not show statistical significance. Furthermore, the abundances of HMGB1 and HMGB1 signaling-associated proteins such as Toll-like receptors (TLR) and NFκB subunits p50, p52, and p65 were also examined in the microarray. These proteins were not significantly different (Figure 7B–G).

To gain a first overview of the significantly altered proteins, they were classified according to cellular component (Table 2), molecular function (Table 3), and biological processes (Table 4) by Gene Ontology analysis. It was found that the highly abundant hnRNPs play a role in regulating gene expression (GO term 10468) and RNA metabolism (GO term 16070).

**Figure 8:**
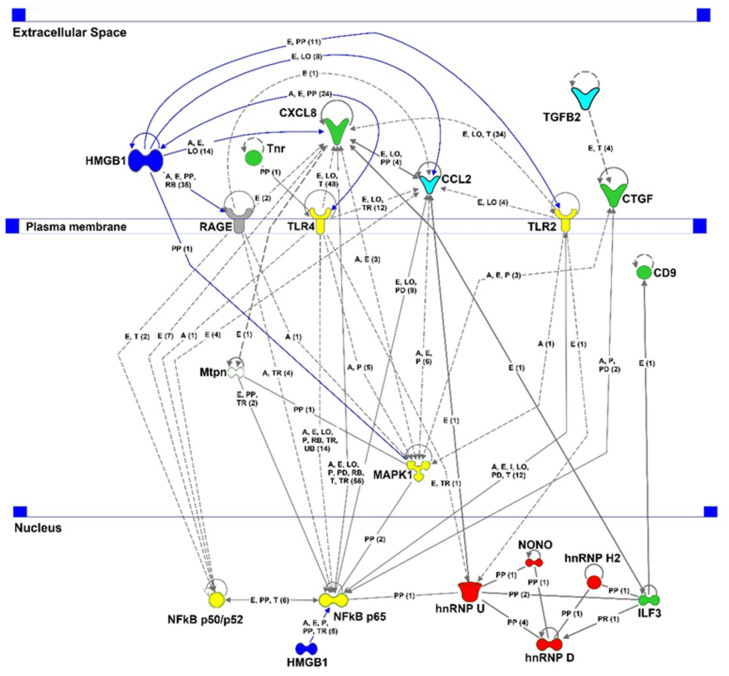
IPA interaction network of selected proteins identified by mass spectrometry or microarray. The significantly downregulated proteins in the Ab-treated group compared with the control group are shown in green; the upregulated proteins are shown in red. Selected proteins identified by microarray or mass spectrometry that were not significantly different in the groups studied are yellow. The proteins shown in grey show considerable interactions with the differentially expressed proteins shown by previous studies. Legend: A = activation; L = proteolysis; P = phosphorylation/dephosphorylation; PP = protein–protein binding; I = inhibition; E = expression; PD = protein–DNA binding; TR = translocation; MB = group/complex membership; solid arrow = direct interaction; dashed arrow = indirect interaction; (count) = number of scientific references on the respective relation; if not indicated differently in the figure, the arrowhead indicates “acts on”.

A protein interaction network was performed using ingenuity pathway analysis to look at protein interactions and to deduce possible treatment mechanisms with an anti-HMGB1 (IPA^®^, Qiagen). The IPA network showed that significantly upregulated (red) and downregulated proteins (green) can be found in various cellular areas, such as nucleus cytoplasm, plasma membrane, or ECM (Figure 8). HMGB1 was manually integrated into this network as a target protein (blue). HMGB1 abundance was not significantly altered in the studied groups. This network shows that HMGB1 can act in the nucleus and extracellular space. In the extracellular space, HMGB1 interacts with receptors TLR-2 and -4 (yellow) and RAGE (gray). This activates MAPK1 (yellow) and NFκB subunits p50, p52, and p65 (yellow). TLR-2 and -4 activate NFκB p65, whereas TLR-4 and RAGE can regulate NFκB p50 or p52 in addition to NFκB p65. These, in turn, regulate the expression of CXCL8, CTGF, and CCL2. TGFβ2 induces the protein and gene expression of CTGF. In addition to HMGB1, Tnr can interact with TLR-4 and promote TLR-4-dependent signaling. NFκB p65 can interact not only with HMGB1 but also with hnRNP U, a protein that interacts with hnRNP D. Together with hnRNP H2, hnRNP D and hnRNP U form a protein complex that mediates RNA metabolism and gene regulation.

## 3. Discussion

HMGB1 is a protein that has been known for a long time; it can act both intracellularly and extracellularly. HMGB1 can act as a DAMP to promote the inflammatory response associated with many neurodegenerative diseases [25,30,31,32]. Actively or passively secreted by dying neurons, it acts as a Toll-like receptor ligand and is involved in RAGE signaling [33,34]. In acute glaucoma models, it stimulated the NLRP3 inflammasome via the activation of NFκB, triggering caspase 8-dependent apoptosis [35,36]. Therefore, the HMGB1 protein has considerable value as a potential biomarker, while indicating its potential as a promising therapeutic target [37]. HMGB1 inhibition was shown to attenuate the inflammatory response in other diseases [38]. In particular, the use of a monoclonal anti-HMGB1 Ab has been shown to be effective in treating central nervous system disorders such as stroke, traumatic brain injury, Parkinson’s disease, or Alzheimer’s disease [39]. The effect of an anti-HMGB1 Ab in a long-term glaucoma model with chronic IOP elevation has not yet been investigated. However, in one of our studies, proteomic analysis of retinal tissue obtained from the eyes of a donors with POAG showed increased HMGB1 expression levels compared with healthy control retinae [40].

Furthermore, in a rat glaucoma animal model, travoprost but not dorzolamide eye drops decreased HMGB1 expression in the retina, despite a successful decrease in IOP with both eyedrops [41]. This indicates that HMGB1 might play a critical role in inflammatory responses in glaucoma disease, which is independent of the elevated IOP [41]. Therefore, the present study aimed to evaluate the neuroprotective effects of the intravitreal injection of an anti-HMGB1 antibody in a glaucoma animal model.

In this glaucoma animal model, the elevated IOP was induced by the thermal occlusion of three different episcleral veins. The reduced venous outflow of the aqueous humor resulted in efficient and reproducible IOP elevation [42]. Since all arteries and one vein remained open, no ischemia in the affected eye was induced. The pressure elevation was moderate to about 1.5 times greater and, thus, could be defined as sub-ischemic, as in other studies [43]. This surgical intervention is minimally invasive and brief when performed by experienced experimenters. Because the observation period started after the stable increase in IOP at 3 weeks and was persistent for 7 weeks, we expected the observable effects to be derived from IOP elevation or the associated consequences, such as the slow progressive loss of RGCs, which is characteristic of glaucoma [44].

The 1.5-fold IOP increase from the baseline level remained significantly elevated until week 10 of the study (*p* < 0.001), which resulted in significant structural changes in the retina. Comparing the eyes of the control animals (IgG OS vs. IgG OD), the RNFL and the RGC density showed a significant decrease by week ten of the study. This is consistent with previous studies that used episcleral vein occlusion to increase IOP, which mimics the pathomechanism of glaucoma [17]. 

The anti-HMGB1 Ab application significantly decelerated the loss of RNFLT and RGC density.

The damage grading in PPD-stained optic nerves could be shifted from severe damage in the IgG OS group, grade four and five, to moderate or minor damage in the anti-HMGB1 Ab-treated group, mainly grade one and two.

For a successful application of the anti-HMGB1 Ab as a potential therapeutic approach, retina functionality must be considered, which can be tested, for example, by ERG [45,46]. We expected a low signal of the PhNR amplitude in the IgG OS compared with the OD due to the significant RGC loss [47]. However, the application of anti-HMGB1 Ab only slightly improved the PhNR amplitude, which was mainly derived from the functionality of the RGCs. The B-wave amplitude was reduced in the IgG OS control compared with the IgG OD, indicating that RGCs and outer retinal layers are involved in neuronal impairment [47]. Previous studies have confirmed these observations from experimental glaucoma animal models in mice and rats [48,49,50,51]. The B-wave amplitude, mainly derived from horizontal and bipolar cells, was remarkably improved after treatment with an anti-HMGB1 Ab. The enhanced B-wave amplitude could also be observed in an animal model of diabetic retinopathy, in which HMGB1 expression was diminished by treatment with siRNA [52].

The anti-HMGB1 Ab treatment revealed clinically measurable neuroprotective effects on structure and function. For a more profound analysis, microarray was used to examine the expression levels of HMGB1 and HMGB1 receptors, TLR-2 and TLR-4. Surprisingly, the anti-HMGB1 treatment did not result in any striking change in expression levels. Therefore, the neuroprotective effect does not appear to occur at this level. It has already been shown that antibody treatments do not necessarily lead to a reduction in the target protein but rather to a blocking of the target protein and, in this way, affect downstream effects [53,54].

**Figure 9:**
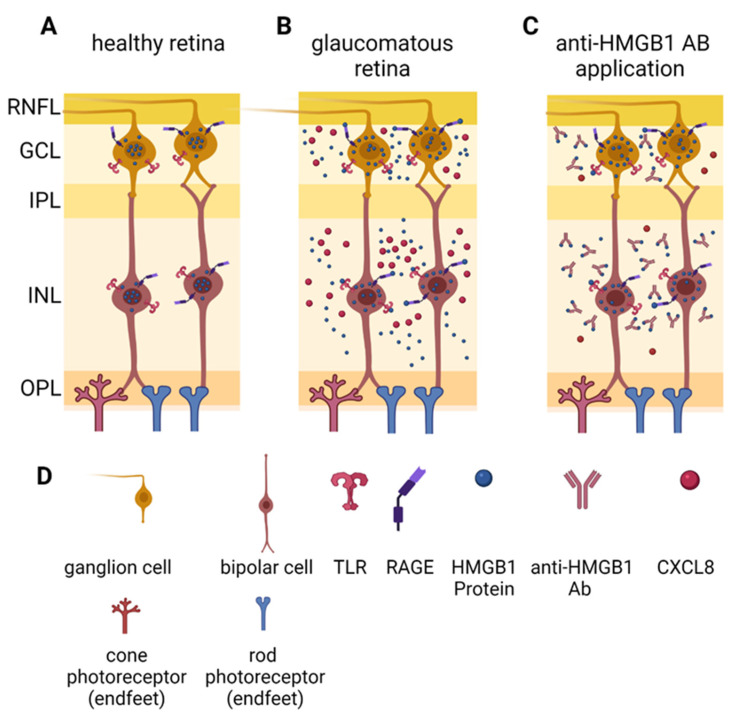
Schematic illustration of a proposed mechanism of anti-HMGB1 Ab application. (**A**) Schematic section of the healthy retina. HMGB1 is mainly located in the nucleus. (**B**) Schematic section of glaucomatous retina. HMGB1 translocates from the nucleus to the cytoplasm. Additionally, HMGB1 is secreted by cells and binds its receptors, such as Toll-like receptors (TLRs) or receptor for advanced glycation endproducts (RAGE). As a result, the expression of cytokines such as CXCL8 is induced and leads to an inflammatory response. (**C**) Glaucomatous retina with anti-HMGB1 Abs. Expression and distribution of HMGB1 is comparable to (**B**). By application of anti-HMGB1 Ab, HMGB1 is mainly captured by the Ab, which suppresses binding to TLRs or RAGE. Thereby, signaling through these receptors is reduced and thus the expression of inflammatory cytokines such as CXCL8 is also reduced, consequently reducing the inflammatory response. Abbreviations: RNFL—retinal nerve fiber layer, GCL—ganglion cell layer, IPL—inner plexiform layer, INL—inner nuclear layer, OPL—outer plexiform layer, CXCL8—C-X-C motif chemokine ligand 8/ interleukin 8 (IL-8). (**D**) Legend for subfigures (**A**–**C**). This figure was created with BioRender.com.

Therefore, we used microarray to investigate the various proteins involved in pro-inflammatory signaling. CXCL8 expression was significantly downregulated in the HMGB1 OS group. Additionally, the mean value of CCL2 expression was notably lower in the anti-HMGB1 Ab-treated group compared with the control group. CXCL8 and CCL2 are pro-inflammatory cytokines that are elevated in various forms of glaucoma [55,56,57,58,59]. In addition, CXCL8 was found to be elevated in an experimental animal model of optic nerve crush [60]. In addition, the IPA interaction network showed that HMGB1 regulates CXCL8 expression via NFκB. As an angiogenesis factor, CXCL8 is involved in new blood vessel formation. CXCL8 is an inflammatory mediator involved in the chemotactic recruitment of leukocytes and granulocytes to the inflamed tissue [55]. Therefore, the decreased abundance of CXCL8 by anti-HMGB1 antibody treatment indicates that retinal stress may have been reduced, which, in turn, could have reduced cytokine levels as well as the recruitment of pro-inflammatory cells. Like CXCL8, CCL2 promotes the expression of pro-inflammatory cytokines, stimulating the inflammatory response [61,62,63]. Thus, the decreased CXCL8 and CCL2 abundance suggest a reduction in the pro-inflammatory environment, possibly achieved by the anti-HMGB1 Ab application (Figure 9).

The anti-HMGB1 Ab injection considerably reduced the connective tissue growth factor (CTGF), CD9, and the TGFβ2 expression. These proteins are regulated by HMGB1 and play an essential role in modulating the ECM. The TGFβ2 protein, in turn, regulates the CTGF abundance [64]. These proteins were found to be elevated in studies with trabecular meshwork cells and the aqueous humor of glaucoma patients [65,66,67]. CTGF and TGFβ2 caused increased ECM production, thus stiffening the tissue, leading to decreased aqueous humor outflow and elevated IOP [68,69]. The function of TGFβ2 and CTGF in the retina, specifically in the experimental glaucoma animal model, is not yet fully understood. In retinal vascular diseases such as diabetic retinopathy and age-related macular degeneration, TGFβ2, CD9, and CTGF are elevated, inducing choroidal and retinal micro vessel development [70,71,72,73]. This could lead to the infiltration of inflammatory cells as described for animal models with renal damage [74]. Therefore, anti-HMGB1 treatment may lead to a reduced stiffness of the retinal tissue and reduced development of retinal micro vessels, conferring neuroprotection in this study.

According to the IPA interaction network, the proteins described above have NFκB as a shared transcription factor, which we also examined in the microarray. The expression of NFκB_p65_ was not altered. However, the mean levels of NFκB_p50_ and NFκB_p52_ were noticeably lower in the HMGB1 OS group compared with the control group, which may indicate the lower expression of downstream genes. Accordingly, microarray analysis showed that HMGB1 treatment alleviated the pro-inflammatory cytokines CXCL8 and CCL2, although the expression level of HMGB1 was not reduced. Therefore, it seems plausible that anti-HMGB1 Ab treatment reduces the pro-inflammatory effect by HMGB1 blockage. As a result, HMGB1 cannot bind to receptors, which could reduce pathway activity. This could explain the reduction in NFκB expression, which results in CXCL8 and CCL2 reduction.

To identify the molecular effects of the anti-HMGB1 Ab application in a larger context, proteomic analyses of the retina were performed by mass spectrometry. The heterogeneous nuclear ribonucleoproteins (hnRNPs) hnRNP D0, hnRNP H2, and hnRNP U were significantly more abundant in the HMGB1 OS group than in IgG OS. HnRNPs are critically involved in nucleic acid metabolism and can be found in the nucleus and cytoplasm [75,76]. HnRNPs are specific for binding mRNA. This interaction allows mRNA to translocate into axons, allowing HnRNPs and mRNA to be detected far from neuronal somas [77].

The spatial regulation of protein synthesis responds to localized stimuli to maintain cellular homeostasis [78,79]. The translated proteins originating from mRNAs localized to axons are essential to facilitate axons’ growth, survival, and injury responses [80]. HnRNP D regulates protein expression by interaction with hnRNP H2 and U to regulate the transcriptional elongation and axon growth [81,82,83]. HnRNP D, H1, and F have been shown to bind HMGB1 mRNA and regulate the translation of HMGB1 mRNA [84]. The HMGB1 protein was shown to promote neurite outgrowth [85,86]. Therefore, HnRNPs D, H1, and U are critical for the localization of HMGB1 mRNA and the protein expression at concentrated sites of need to cause neuronal maintenance and branching, which may represent a neuroprotective mechanism for the retina in our study.

The importance of hnRNPs has become particularly apparent in recent years, as a lower expression of hnRNPs has been associated with neurodegeneration. For example, hnRNP H regulates protein kinase Cα (PRKCα) expression. Low PRKCα expression predisposes individuals to multiple sclerosis [87]. Nuclear reductions in hnRNP D are associated with human skeletal muscle-wasting diseases [88,89]. HnRNP D0 and U mutations can increase their affinity for tau protein binding. Both nuclear reduction and mutations of hnRNPs can promote amyotrophic lateral sclerosis [90,91]. Other hnRNPs such as hnRNP A1 or C1, C2, and K are also involved in axon development or regeneration and neurodegeneration [92,93]. For example, HnRNP K knockdown disrupted optic nerve regeneration in *Xenopus laevis* [94]. In addition to the neurodegenerative role of hnRNPs, a protective role of hnRNP A18 in genotoxic stress has also been sporadically described [95].

Information on hnRNPs in glaucoma is scarce. In a previous study, our group identified hnRNPs U and Q in human retinae from donor eyes [40]. These were less abundant in the retinae of POAG patients than in the control retinae, confirming the potential neuroprotective functions of hnRNPs.

In addition to hnRNPs, myotrophin (Mtpn) was also found to be significantly more abundant in the HMGB1 OS group compared with the IgG OS group. Mtpn inhibits the F-actin capping protein CP to prolong actin cytoskeleton formation [96]. HnRNPs form a translational complex, which interacts with the actin cytoskeleton, indicating retinal restructuring or even possible axon growth, as previously described [96]. Different cell culture experiments and analyses in a glaucoma animal model described increased cytoskeletal stability as a neuroprotective mechanism [16,17].

In addition to intracellular proteins, extracellular proteins were also identified by MS. Tenascin-R (Tnr), a protein belonging to the tenascin protein family [97,98], was found to be significantly less abundant in the HMGB1 OS group compared with the IgG OS group. Tnr is restricted to expression in neuronal tissues such as the central nervous system or retinal neurons [99,100] and interacts with extracellular matrix (ECM) proteins such as fibronectin and plasma membrane-bound receptors such as TLR-4 [101,102]. Tnr is continuously expressed under physiological conditions. Tnr is upregulated under inflammatory conditions, promoting the expression of pro-inflammatory cytokines such as TNF-α and CXCL8 [102,103,104]. Tnr restricts the modulation of motor neurons and the development of axonal regeneration in mice and model organisms and is thus responsible for neurodegenerative processes [99,103,105,106,107,108]. It has been described that tenascin expression is elevated in POAG patients [109,110]. A significantly lower expression of Tnr in the anti-HMGB1 antibody-treated group might indicate a lower inflammatory level in the retina during IOP elevation. A lower tenascin expression was beneficial in experimental studies, such as in an experimental autoimmune glaucoma model [111,112]. In turn, neuroprotective effects were demonstrated by Tnr-modulated microglia [100]. This indicates the potential involvement of the ECM in the HMGB1-induced neuroprotective effect.

Although the presented study shows a new approach for an IOP-independent glaucoma therapy, some study limitations should be pointed out. A single animal model cannot capture all features of a complex disease such as glaucoma. Furthermore, animal studies are always subject to limited tissue. Thus, this study investigated proteins and pathways directly related to HMGB1. Further studies should confirm these results in depth and reproduce them in other animal models, as each model represents different disease features. 

In conclusion, this study showed that an anti-HMGB1 Ab improved the survival of RGCs and axons in a chronic elevated-IOP glaucoma animal model compared with a control. These data demonstrate the neuroprotective effect of anti-HMGB1 Ab application by reducing pro-inflammatory signaling, leading to improved RGC survival. However, further studies need to explore this to elucidate the potential of targeting HMGB1 as a promising therapeutic target. The anti-HMGB1 Ab could be applied in synergistic Ab therapy for future therapeutic approaches to affect different signaling pathways simultaneously.

## 4. Materials and Methods

### 4.1. Animals and Anesthesia

All experiments in this study were performed according to the Association for Research in Vision and Ophthalmology (ARVO) guidelines. The methods used were reviewed and approved by the National Investigation Office of the State of Rhineland-Palatinate in Koblenz, Germany, concerning the legal requirements and the ethical acceptability of the experimental project (23 177-07/G15-1-053).

In this study, we used female Sprague Dawley rats of the breeder Charles River (Sulzfeld, Germany) of seven weeks of age or approximately 190 g body weight. All examinations, except the IOP measurements, were performed with appropriate anesthesia. For this purpose, 0.7 mg ketamine (Ketamine Inresa, 50 mg/mL, Inresa Arzneimittel GmbH, Freiburg, Germany) and 0.25 mg medetomidine (1 mg/mL, Dorbene vet., Zoetis Deutschland GmbH, Berlin, Germany) per kg body weight were used for the episcleral vein occlusion (EVO) in a mixed syringe. For the other experiments, optical coherence tomography (OCT) and electroretinogram (ERG), only 0.135 mL/kg of medetomidine was injected intramuscularly. The anesthetic effect was antagonized by intramuscular injection of 0.2 mL/kg atipamezole (Alzane^®^, 5 mg/mL, Zoetis, Zoetis Deutschland GmbH, Berlin, Germany). EVO was performed unilaterally for chronic IOP elevation in the left eye (OS); the right eye (OD) served as an internal control.

### 4.2. Experimental Design 

All animals were kept in a temperature- and humidity-controlled room with a 12 h light–dark cycle. The animals had ad libitum access to water and food. Each animal underwent the following regular examinations: IOP measurements, OCT, and ERG analysis. The animals with stably elevated IOP were examined and divided into two groups depending on the antibody injected intravitreally in OS. The control group (IgG OS, *n* = 5) received 50 µg of a mouse-derived control antibody with no known specificity (Mouse IgG2b Isotype Control, 02-6300, Thermo Fisher Scientific, Rockford, IL, USA). The treatment group (HMGB1 OS, *n* = 6) received 50 µg of a mouse-derived anti-HMGB1 antibody (1F3, NBP2-25148, Novus Biologicals, Littleton, CO, USA). The OD did not receive an intravitreal injection (IVI). IVI was given immediately after the stable elevation of IOP, three weeks after EVO. Ten weeks after EVO, the animals were sacrificed by carbon dioxide overdose.

### 4.3. Intraocular Pressure (IOP) Monitoring by Rebound Tonometry

A TonoLab rebound tonometer (iCare, Espoo, Finland) designed explicitly for rodents was used to measure IOP. The IOP is determined by the rebound speed of the tonometer probe, i.e., the harder the surface, the higher the intraocular pressure, which prevents the probe from deforming the corneal surface, which in turn provides a shorter rebound time and a higher rebound speed. For correct measurements, both the animal and the TonoLab must be held horizontally. For this purpose, the experimenter held the TonoLab, and an assistant held the animal to be measured. After initial but unrecorded measurements, ten consecutive correct measurements were taken and documented. Outliers caused by the device or the animal’s movement were excluded and not recorded. Measurements were taken weekly between 8 am and 1 pm.

### 4.4. Chronic IOP Elevation by Episcleral Vein Occlusion (EVO)

**Figure A1:**
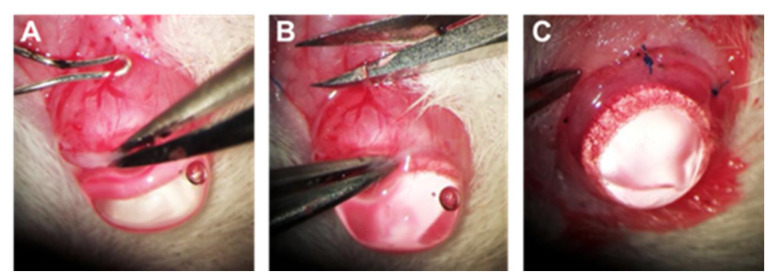
Episcleral vein occlusion on a rat eye. The EVO operation has several intermediate steps: (**A**) Incision of the conjunctiva, exposure of the episcleral veins and closure of the truncti by thermo-occlusion. (**B**) Dissection of the empty vessel to prevent bypass formation. (**C**) Closure of the incision by surgical knots.

After systemic anesthesia, the OS was topically anesthetized by one or two drops of 4 mg/mL oxybuprocainhydrochloride (Novesine^®^ 0.4% Eyedrops, OmniVision^®^, OmniVision GmbH, Puchheim, Germany). Only the IOP of the animal’s left eye was chronically increased by episcleral vein occlusion, as described by Shareef et al. (1995) [42]. For this purpose, the conjunctiva was incised in the area from the nasal superior to the temporal. Connective tissue was removed to expose the episcleral veins. Subsequently, three of the four episcleral veins present were thermally occluded by a cautery before branching (Figure A1A). To prevent the development of the bypass veins, the trunci of the veins were cut and checked to see whether the blood flow was interrupted (Figure A1B). Finally, the conjunctiva was readapted, and the wound was closed with surgical knots (Figure A1C). To protect the cornea, Bepanthene ophthalmic ointment was applied to the cornea of the OD, and Corneagel was applied to the cornea of the OS before the surgery, as it is transparent and facilitates work on the eye. Three drops of novamine sulfonic acid (500 mg/mL Metamizole, Ratiopharm GmbH, Ulm, Germany) relieved possible postoperative pain.

### 4.5. Intravitreal Injection of Antibodies

The antibodies were applied intravitreally in the left eye as soon as the animals showed a stably elevated IOP. For this purpose, 50 µg of the antibody in a volume of 3.5 µL, considered the optimal volume for intravitreal injection, was applied using a 10 µL Hamilton syringe (Sigma Aldrich, Steinheim, Germany) with a 33-gauge needle [113,114]. Special care was taken not to touch the lens or retina or disturb the retinal blood supply. The needle remained in the same position for at least 30 s to prevent an outflow of the antibody and allow distribution in the vitreous. In vivo, OCT was used to evaluate the effect of the injection on retinal integrity. No noticeable differences between the experimental groups and the OS or OD of the animals could be detected.

### 4.6. RNFL Thickness Measurement by Optical Coherence Tomography (OCT)

The retinal nerve fiber layer thickness (RNFLT) was observed by spectral-domain OCT (SD-OCT, Spectralis OCT + HRT, Heidelberg Engineering GmbH, Heidelberg, Germany), as described previously [17]. For this purpose, the animals’ eyes were dilated after anesthesia by topical application of tropicamide (Mydriaticum, Pharma Stullen, Stullen, Germany). Local anesthesia of the eye was performed by topical application of Novesine. Improving the recording quality, a contact lens suitable for rodents (Cantor + Nissel, Northamptonshire, England, PMMA 2.70/5.20, the radius of curvature of the central optic zone: 2.70 mm; diameter: 5.20 mm) was applied to the eye to be examined. The device had to be adapted for the examination of animals. A custom-made platform was used to place the animals horizontally. The corneal radius was set to 7.7, and the reference arm and focus were adjusted for each animal. A circular B-scan around the optic disc with a 12° diameter was acquired for each acquisition, consisting of 1536 A-scans. The signal-to-noise ratio was improved by real-time eye-tracking and averaging the 100 received frames. Eye-tracking during infrared imaging of the fundus allowed RNFLT to be observed throughout the study period and therefore to be analyzed both cross-sectionally and longitudinally. The B-scan was analyzed using Heidelberg Eye Explorer software. The analysis was performed using an algorithm developed for humans and therefore had to be manually adjusted for each image, as described in [17]. The software divides the retinal surface into different sectors: temporal superior (TS), temporal (T), temporal inferior (TI), nasal superior (NS), nasal (N), and nasal inferior (NI). For each area, the RNFLT was calculated as well as an overall average of all regions. Baseline examinations were performed before EVO (week 0); further investigations were performed four weeks after EVO (week 4), as well as seven and ten weeks after EVO (week 7, week 10).

### 4.7. Photopic Ganzfeld Electroretinogram (ERG)

**Figure A2:**
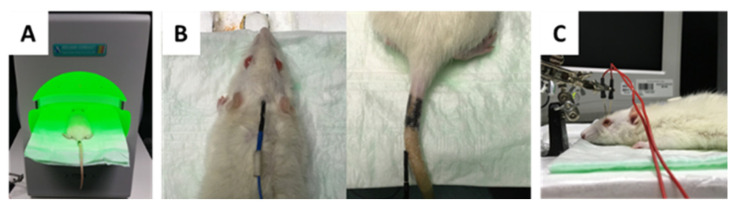
Setup of the photopic Ganzfeld ERG. (**A**) For photopic Ganzfeld ERG, a green background light is permanently present during the measurement, and the animal under investigation is positioned horizontally on a custom-made platform. (**B**) Two reference electrodes are placed on the animal, one on the head centered between the ears, and one on the tail, near the root of the tail. (**C**) The measuring electrodes (gold ring electrodes) are applied to the cornea with light pressure. Methocel was applied between the electrode and the cornea.

Animals were anesthetized, and pupils dilated using Mydriaticum before the examination. In addition, the eye was topically anesthetized with Novesine. For good contact and to prevent injury to the cornea, it was covered with 2% Methocel (OmniVision, Puchheim, Germany). After that, the animal was positioned horizontally on a platform made for this device (Figure A2A). The animal was then connected to four electrodes (Roland Consult, Brandenburg, Germany): two reference electrodes (steel needle electrodes) were attached to the head and tail (Figure A2B), and two measuring electrodes (gold ring electrodes, diameter 4 mm) were applied to the cornea with medium pressure (Figure A2C). As described, the photopic Ganzfeld ERG was recorded with the RETI system (Roland Consult, Brandenburg, Germany) [115]. Green background light with a light intensity of 40 cd∙s∙m^−2^ was used to imitate daylight and saturate the cones. The sequence of white light stimuli with intensities of −0.15, 0.23, 0.61, 0.99, and 1.37 log10 cd∙s∙m^−2^ was used to measure retinal functionality. The acquisition time after each stimulus was 512 ms. A total of 25 ERGs were correctly recorded for each intensity, and the mean value excluded artifacts automatically during recording from the calculation. The frequency of the successive stimuli was 0.33 Hz. The correct fit of the electrodes was ensured by measuring the impedance and looking at the baseline signal. Raw data were exported from the system after measurements and further processed for use. B-wave amplitude and photopic negative response (PhNR) amplitude were used for evaluation.

### 4.8. Immunofluorescence Staining of Retinal Ganglion Cells of Retinal Flat Mounts

Immediately after the sacrifice of the animals, the two eyes were enucleated and transferred to PBS (1 × PBS, Gibco, Thermo Fisher, Waltham, MA, USA). The eye was incised circumferentially at the corneal limbal junction so that the cornea, lens, and loose vitreous could be carefully removed. Subsequently, the retina and the sclera were separated. The retina was then slightly incised in four places and ultimately transferred to a filter paper and further processed in fresh PBS by separating the remaining glass body from the retina as best as possible. Subsequently, the retina was cut into four quarters, two-quarters of which were again detached from the filter and transferred into Eppendorf tubes, frozen in liquid nitrogen, and stored at −80 °C for later proteomic analysis. At the same time, the remaining quarters were transferred to 4% paraformaldehyde (PFA, Histofix, Roth, Karlsruhe, Germany) and thus fixed for 30 min. The retina was then washed with PBS and dehydrated overnight in 30% saccharose solution in the refrigerator at 4 °C. Finally, the retina was frozen in liquid nitrogen and stored at −20 °C for further processing. After thawing, the retina was washed three times with PBS for ten minutes. In preparation for staining, the retina was incubated in a blocking solution of 0.3% Triton X-100 to permeabilize the cell membranes and 10% FCS to block for about 2 h. The brain-specific homeobox/POU domain protein 3 A (Brn3a) was used to stain retinal ganglion cells (RGCs) [116]. The primary goat anti-Brn3a polyclonal antibody (1:125, C-20, 31,984 Santa Cruz Biotechnology, Santa Cruz, CA, USA) was incubated in the blocking solution described above at 4 °C overnight. The retina was then washed with PBS and incubated with the secondary anti-goat antibody coupled with AlexaFluor568 (1:400, #A-11057, Invitrogen, Carlsbad, CA, USA) in a blocking solution for 2 h. The retina was washed and finally transferred to a slide with the RGCs facing up. The retina was mounted with Vectashield Mounting Medium (VECTASHIELD Antifade Mounting Medium, H-1000, Vector Laboratories, Burlingame, CA, USA). Finally, the retina was covered with a cover glass. Images of Brn3a-positive cells were obtained with the Eclipse TS 100 fluorescence microscope (Nikon, Yurakucho, Tokyo, Japan) with a DS-Fi1-U2 digital microscope camera (pixel pitch 3.5 µm, Nikon), an ELWD 20×/0.45 S Plan Flour Ph1 ADM objective (Nikon) and NIS Elements recording software (Nikon, version 4.10 64 bit). The retina was divided into three regions depending on the distance to the ONH, peripapillary, mid-peripheral, and peripheral, and three images of each area were taken. Thus, each retina quarter was captured with nine images with a size of 0.142 mm^2^. The RGC count was determined using a semi-automatic macro of the software ImageJ (http://rsb.info.nih.gov/ij/ (accessed on 3 February 22), NIH, Bethesda, MD, USA). The following steps were performed when using the macro: (1) convert to 8-bit, (2) subtract background, rolling ball radius 120, (3) set auto threshold automatically, and (4) run “nucleus counter” with smallest 400 and largest 7000. Analysis was performed in a blinded manner.

### 4.9. p-Phenylenediamine (PPD) Staining of Optic Nerve Cross-Sections

After enucleation of the eyes, the optic nerve was separated near the optic chiasm. The piece, which included the optic nerve head, was immediately transferred to a 3% glutaraldehyde solution for fixation. After staining with p-phenylenediamine (PPD), the optic nerves were cut into semi-thin vertical sections about 400 nm thick, transferred to a microscope slide, and covered with a coverslip. An overview image of the optic nerve was acquired. Seven images of each optic nerve (IgG OS *n* = 4, HMGB1 *n* = 6) were taken to document different regions of the nerve. The sections were imaged with an upright microscope and a 100× immersion oil objective (Olympus Vanox-T AH-2; Olympus SPlan 100×/1.25 oil, 160/0.17, Olympus Deutschland GmbH, Hamburg, Germany). For analysis, completely collapsed axons were counted and summed over the seven recordings for each nerve. For the gradation of optic nerve damage, 5 grades were equally divided depending on the maximum number of collapsed axons [117].

### 4.10. Discovery Proteomics

Protein extraction from the collected retinal pellets (HMGB1 OS *n* = 5, IgG OS *n* = 5) was performed as described in previous publications using the tissue protein extraction reagent buffer (T-PER, Thermo Fisher Scientific, Rockford, IL, USA). After protein homogenization, a buffer exchange was performed using 3 kDa Amicon^®^ ultra centrifugal filters (Merck Millipore, Darmstadt, Germany) to enrich and dissolve the proteins in LC-MS-grade water. Subsequently, the protein concentration of each sample was determined using a BCA assay kit (Thermo Fisher Scientific, Rockford, IL, USA) and measured with the Multiscan Ascent photometer (Thermo Fisher Scientific, Rockford, IL, USA) at 570 nm. Then, a protein amount of 10 µg of each sample was evaporated to dryness in the SpeedVac (Eppendorf, Darmstadt, Germany) for 30 min at 30 °C. The in-solution trypsin digestion and the peptide purification before the LC-MS analysis were performed as already described in previous publications [118]. The liquid chromatography–mass spectrometry (LC-MS) measurements were performed with a hybrid linear ion trap–Orbitrap MS system (LTQ Orbitrap XL, Thermo Fisher Scientific, Rockford, IL, USA) online coupled with the EASY-nLC 1200 system (Thermo Fisher Scientific, Rockford, IL, USA). The purified and tryptic digested samples were dissolved in 80 µL of 0.1% formic acid (FA, dissolved in H_2_O, solvent A), and 2 µL of each sample (0.125 µg/µL) was injected into the system per run. The flow rate was set to 300 nl/min, and the peptides were separated using a PepMap C18 column system (75 µm × 500 mm; Thermo Fisher Scientific, Rockford, IL, USA). For peptide elution, solvent B (0.1% FA dissolved in Acetonitrinle, ACN) was used. The gradient for the peptide elution was set to 200 min as follows: 5–30% B (0–160 min), 30–100% B (160–180 min), and 100% B (180–200 min).

The LTQ Orbitrap operated in the positive ionization mode and data-dependent acquisition (DDA) mode: a high-resolution scan (m/z 300 to 2000) was performed in the Orbitrap with a resolution of 30,000 at 400 m/z. The automatic gain control was set to 1∙10^6^ ions. For internal calibration, the lock mass was set to 445.120025 m/z (poly-dimethyl cyclosiloxane). The dynamic exclusion mode was enabled with the following settings: repeat count = 1, repeat duration = 30 s, exclusion list size = 100, exclusion duration = 300 s, and exclusion mass width = ±20 ppm. The five most intense precursor ions were selected for collision-induced dissociation (CID) fragmentation in the ion trap using a normalized collision energy of 35%. The LC-MS raw data were analyzed with the bioinformatics software MaxQuant v. 1.6.17 (Max Planck Institute for Biochemistry, Martinsried, Germany) for protein identification and quantification. Tandem MS spectra were searched against the SwissProt database with the taxonomy *Rattus norvegicus* (date: 4 May 21, entries: 8131 sequences) and the following settings: peptide mass tolerance of ±30 ppm, fragment mass tolerance of ±0.5 Da, tryptic cleavage, a maximum of two missed cleavage, carbamidomethylation as fixed modification, and acetylation (protein N-terminal) as well as oxidation as variable modifications. All protein identifications were filtered with a false discovery rate (FDR) < 1%.

### 4.11. Analysis and Bioinformatics of MS Data

Statistical analysis of the MaxQuant generated output data (“proteins.txt”) was performed using the software program Perseus, version 1.6.15.0 (Max Planck Institute of Biochemistry, Martinsried, Germany). For analysis, the LFQ intensities were log_2_ transformed. Subsequently, the data were filtered for contaminants. Hits only ‘identified by side’ were also excluded. The identification of the proteins was based on at least two peptides, which must have been detected in all replicates of the study group. Missing intensity values were imputed by random numbers received from the normal distribution. To analyze whether the proteins changed significantly, a two-sided Student’s *t*-test was performed. Protein changes with *p*-values < 0.05 were identified as statistically significant.

The mass spectrometry proteomics data have been deposited to the ProteomeXchange Consortium via the PRIDE partner [119] repository with the dataset identifier PXD031987.

### 4.12. Antibody-Based Microarray

Customized arrays were produced in-house using a non-contact array printer (SciFLEXARRAYER S3, Scienion, Berlin, Germany) [120]. The antibodies selected for the microarray are listed in the Appendix A (Table A1). Antibodies were prepared in a dilution of 0.25–0.5 mg/mL in PBS and transferred to a 384-well plate. The plate was centrifuged at 800× *g* at 4 °C for 2 min and then placed on the plate holder of the array spotter. The spotter settings were then adjusted, and spotting was performed at 60% humidity in the spotter chamber. Antibodies were spotted in triplicate on nitrocellulose-coated glass slides (AVID Oncyte, 16 Pad NC slides, Grace Bio-Labs, Bend, OR, USA). The slides were stored at 4 °C in the refrigerator for later use. Retinal tissue was used for microarray analysis. Proteins were isolated as described in the ‘Discovery Proteomics’ section of this paper, and protein concentration was determined by BCA assay. For sample preparation, 10 µg of each sample was diluted to generate 100 µL of the sample at a dilution of 1 mg/mL in PBS. A total of 1 µL of DyLight 650 NHS Esther (Thermo Scientific, Waltham, MA, USA) was added to each sample. Pure PBS buffer with labeling dye was used as a negative control. All samples were incubated for 1 h at room temperature in the dark. Then, 10 µL of the quenching solution (1M Tris-HCl pH 8.8) was added to quench the unbound dye. The solution was incubated for 30 min at room temperature in the dark. According to the manufacturer’s instructions, unbound dye and buffers that could negatively affect array hybridization were removed using Zeba desalting columns (Thermo Fischer). The purified samples were frozen at −20° and protected from light for later use.

Before incubation with the sample, the array slides were incubated with a blocking buffer (Super G, Grace Bio-Labs, Bend, OR, USA) for 1 h at room temperature on an orbital shaker. The slides were then washed four times with PBST (PBS with 0.5% Tween 20). Subsequently, the slides were incubated with the purified samples for 2 h on an orbital shaker. After that, the slides were washed two times each with PBST and two times with ultrapure water. Slides were subsequently dried in a vacuum centrifuge. Array images were acquired as 16-bit TIF files using a CCD camera-based microarray reader (SensoSpot Microarray Analyzer, Sensovation, Radolfzell, Germany). The image analysis software Imagene (Imagene 5.5, BioDiscovery Inc., Los Angeles, CA, USA) was used to quantify spot intensities. Poor-quality spots were manually flagged and excluded from the analysis.

### 4.13. Microarray Data Processing

The microarray data were pre-processed: The local background signal was subtracted from the measured spot intensity to obtain a net signal intensity. Negative net intensities were treated as missing data. The signal intensities of the negative control were also subtracted from the net intensities to account for the non-specific binding of excess dye. Finally, the intensity of the triplicate spots was averaged to obtain a mean signal intensity used for data analysis.

Gene Ontology enrichment analysis was performed with the Cytoscape software (Cytoscape, version 3.8.2) using the BiNGO plugin (BiNGO version 3.0.3). For this purpose, the significantly altered proteins analyzed by MS and the proteins analyzed by microarray were included. The protein–protein interaction network was formed by ingenuity pathway analysis (IPA, version v.01-04, Qiagen, Venlo, The Netherlands) using the significantly altered proteins.

### 4.14. Statistical Analysis and Image Processing

The data presented are expressed as mean ± standard deviation (SD). Statistical analysis was performed using Statistica version 13 software (Dell Inc. Round Rock, TX, USA). Differences in experimental groups were analyzed by one-way analysis of variance followed by Tukey’s honest significant difference (HSD) post hoc test to compare multiple groups with unequal N. For the statistical analysis of the IOP data, the two-tailed Student’s *t*-test was used to compare two groups for a given time point. Graphs were conducted using GraphPad Prism v.7 (GraphPad Software Inc., San Diego, CA, USA).

### 4.15. Ethical Approval

The national investigation office approved all experimental protocols in Koblenz, Germany (23-177-07/G 15-1-053). The methods were carried out following the relevant guidelines and regulations.

## Figures and Tables

**Table 1:** Summary of OCT analysis including RNFLTs of the IgG OS and HMGB1 groups and their differences.

Experimental Group	RNFLT for the Indicated Regions (%)						
	AV	NS	N	NI	TS	T	TI
HMGB1 OS	94.8	91.9	97.6	92.1	90.3	97.1	99.4
IgG OS	77.3	68.9	69.8	72.4	72.6	81.6	87.1
HMGB1 OS–IgG OS	17.5	22.5	27.8	19.7	17.7	15.5	12.3

**Table 2:** Gene Ontology analysis. Sorted by cellular component.

GO Terms	5623	5737	5634	5739	5886	30529	5576
GO description	Cell	Cytoplasm	Nucleus	Mitochondrion	Plasma membrane	Ribonucleo-protein complex	Extracellular region
Frequency	24/25 96.0%	20/25 80.0%	9/25 36.0%	7/25 28.0%	6/25 24.0%	5/25 20.0%	3/25 12.0%
Genes	MTPN, FDPS, GPR37, NONO, IDH2, HNRNPU, ABAT PSMA7, CTGF, NAP1L4, CALB2, ATP5B, SLC25A18, ILF3, IMPA1, C1QBP, RPL14, HNRNPD, TNR, CD9, HNRNPH2, RBBP7, PPID	MTPN, FDPS, GPR37, IDH2, HNRNPU, ABAT, PSMA7, CTGF, NAP1L4, ATP5B, SLC25A18, ILF3, IMPA1, C1QBP, RPL14, HNRNPD, CD9, HNRNPH2, PPID	ILF3, C1QBP, NONO, HNRNPD, HNRNPU, HNRNPH2, RBBP7, PSMA7, NAP1L4	ATP5B, FDPS, SLC25A18, ILF3, C1QBP, IDH2, ABAT	CALB2, ATP5B, GPR37, C1QBP, CD9, CTGF	ILF3, RPL14, HNRNPD, HNRNPU, HNRNPH2	CXCL8, TNR, CTGF

**Table 3:** Gene Ontology analysis, molecular function.

GO Terms	5515	166	3676	3723
GO description	Protein binding	Nucleotide binding	Nucleic acid binding	RNA binding
frequency	21/27 77.7%	7/27 25.9%	6/27 22.2%	6/27 22.2%
Genes	MTPN, CXCL8, NONO, PDXP, HNRNPU, ABAT, PSMA7, CTGF, NAP1L4, ATP5B, ILF3, IMPA1, C1QBP, RPL14, HNRNPD, TNR CD9, HNRNPH2, RBBP7, PPID	ATP5B, NONO, IDH2, HNRNPD, HNRNPU, HNRNPH2, GTPBP1	ILF3, NONO, RPL14, HNRNPD, HNRNPU, HNRNPH2	ILF3, NONO, RPL14, HNRNPD, HNRNPU, HNRNPH2

**Table 4:** Gene Ontology analysis. Categorized by biological processes.

GO Terms	65007	8152	50896	23052	10468	6950	23060	16070
GO description	Biological regulation	Metabolic process	Response to stimulus	Signaling	Regulation of gene expression	Response to stress	Signal transmission	RNA metabolic process
Frequency	16/26 61.5%	15/26 57.6%	11/26 42.3%	9/26 34.6%	7/26 26.9%	7/26 26.9%	7/26 26.9%	5/26 19.2%
Genes	MTPN, CXCL8, NONO, HNRNPU, ABAT, GTPBP1, PSMA7, CTGF, ATP5B, ILF3, IMPA1, HNRNPD, TNR, CD9, RBBP7	MTPN, FDPS, NONO, IDH2, HNRNPU, ABAT, PSMA7, CTGF, ATP5B, IMPA1, RPL14, HNRNPD, HNRNPH2, RBBP7, PPID	MTPN, CXCL8, IMPA1, C1QBP, NONO, PDXP, CD9, ABAT, RBBP7, GTPBP1, CTGF	CXCL8, GPR37, IMPA1, TNR, CD9, ABAT, GTPBP1, CTGF	MTPN, ILF3, NONO, HNRNPD, HNRNPU, RBBP7, CTGF	MTPN, CXCL8, NONO, CD9, ABAT, RBBP7, CTGF	CXCL8, IMPA1, TNR, CD9, ABAT, GTPBP1	NONO, RPL14, HNRNPD, HNRNPU, HNRNPH2

## Data Availability

Not applicable.

## Appendix A

**Table A1:** Antibodies used for antibody-based microarray.

Antigen	Manufacturer	Order Number
CCL2	Sigma-Aldrich	HPA019163
CD9	Thermo Fisher Scientific	MA1-80307
CTGF	Sigma-Aldrich	HPA031075
CXCL8	Sigma-Aldrich	HPA057179
HMGB1	Novus Biologicals	H00003146-M02
NFkB_p50_	Santa Cruz	sc-166588
NFkB_p52_	Santa Cruz	sc-7386
NFkB_p65_	Santa Cruz	sc-514451
TGFβ2	Sigma-Aldrich	SAB1409735
TLR-2	Abcam	ab13855
TLR-4	Abcam	ab13556
